# Dual-type deep learning-based image reconstruction for advanced denoising and super-resolution processing in head and neck T2-weighted imaging

**DOI:** 10.1007/s11604-025-01756-y

**Published:** 2025-03-05

**Authors:** Noriyuki Fujima, Yukie Shimizu, Yohei Ikebe, Hiroyuki Kameda, Taisuke Harada, Nayuta Tsushima, Satoshi Kano, Akihiro Homma, Jihun Kwon, Masami Yoneyama, Kohsuke Kudo

**Affiliations:** 1 Department of Diagnostic and Interventional Radiology, Hokkaido University Hospital, N14 W5, Kita-Ku, Sapporo, 0608638 Japan; 2 Department of Diagnostic Imaging, Graduate School of Medicine, Hokkaido University, N15 W7, Kita-Ku, Sapporo, Hokkaido 060-8638 Japan; 3 Center for Cause of Death Investigation, Faculty of Medicine, Hokkaido University, N15 W7, Kita-Ku, Sapporo, Hokkaido 060-8638 Japan; 4 Faculty of Dental Medicine Department of Radiology, Hokkaido University, N13 W7, Kita-Ku, Sapporo, Hokkaido, 060-8586 Japan; 5 Department of Otolaryngology-Head and Neck Surgery, Faculty of Medicine and Graduate School of Medicine, Hokkaido University, N15 W7, Kita Ku, Sapporo, 060-8638 Japan; 6 Philips Japan, 3-37 Kohnan 2-Chome, Minato-Ku, Tokyo, 108-8507 Japan; 7 Clinical AI Human Resources Development Program, Faculty of Medicine, Hokkaido University, N15 W7, Kita-Ku, Sapporo, Hokkaido 060-8638 Japan; 8 Global Center for Biomedical Science and Engineering, Faculty of Medicine, Hokkaido University, N14 W5, Kita-Ku, Sapporo, Hokkaido 060-8638 Japan

**Keywords:** Head and neck, MRI, Deep learning reconstruction, Super-resolution

## Abstract

**Purpose:**

To assess the utility of dual-type deep learning (DL)-based image reconstruction with DL-based image denoising and super-resolution processing by comparing images reconstructed with the conventional method in head and neck fat-suppressed (Fs) T2-weighted imaging (T2WI).

**Materials and methods:**

We retrospectively analyzed the cases of 43 patients who underwent head/neck Fs-T2WI for the assessment of their head and neck lesions. All patients underwent two sets of Fs-T2WI scans with conventional- and DL-based reconstruction. The Fs-T2WI with DL-based reconstruction was acquired based on a 30% reduction of its spatial resolution in both the x- and y-axes with a shortened scan time. Qualitative and quantitative assessments were performed with both the conventional method- and DL-based reconstructions. For the qualitative assessment, we visually evaluated the overall image quality, visibility of anatomical structures, degree of artifact(s), lesion conspicuity, and lesion edge sharpness based on five-point grading. In the quantitative assessment, we measured the signal-to-noise ratio (SNR) of the lesion and the contrast-to-noise ratio (CNR) between the lesion and the adjacent or nearest muscle.

**Results:**

In the qualitative analysis, significant differences were observed between the Fs-T2WI with the conventional- and DL-based reconstruction in all of the evaluation items except the degree of the artifact(s) (*p* < 0.001). In the quantitative analysis, significant differences were observed in the SNR between the Fs-T2WI with conventional- (21.4 ± 14.7) and DL-based reconstructions (26.2 ± 13.5) (*p* < 0.001). In the CNR assessment, the CNR between the lesion and adjacent or nearest muscle in the DL-based Fs-T2WI (16.8 ± 11.6) was significantly higher than that in the conventional Fs-T2WI (14.2 ± 12.9) (*p* < 0.001).

**Conclusion:**

Dual-type DL-based image reconstruction by an effective denoising and super-resolution process successfully provided high image quality in head and neck Fs-T2WI with a shortened scan time compared to the conventional imaging method.

## Introduction

T2-weighted imaging (T2WI) is one of the most important sequences in magnetic resonance imaging (MRI) for noninvasive evaluations of the head and neck, as it allows for an overview of the region, lesion detection, the differentiation of diagnoses, and an assessment of the lesion extent [1–4]. Clinically, a fat-suppressed (Fs) technique is often used simultaneously with T2WI for clear lesion detectability by suppressing fat’s high signal around the lesion in T2WI. However, high-spatial-resolution imaging is often required for evaluations of the head and neck due to the complex small anatomical structures of these parts of the body. In addition, image acquisition of the head and neck is sometimes problematic due to the B0 and B1 field inhomogeneity caused by the complicated anatomical shapes and/or the presence of air and any metallic substance [5]. The signal-to-noise ratio (SNR) in head and neck MRI is, thus, frequently insufficient for image evaluation. The acquisition of images with averaging of a larger number of multiple signals may achieve high SNRs, but such scanning needs a long acquisition time, which often results in patient motion and consequently poor image quality.

In most MRI acquisition methods, parallel imaging is typically used as the conventional and standard acceleration technique to shorten the scan time. Advanced techniques such as compressed sensing (CS) and deep learning (DL) image reconstruction are now well developed and used in clinical settings [6–10]. Even with such advanced techniques, however, a long scan time has been required in acquisitions with sufficiently high spatial resolution for evaluations of the head and neck. A DL-based image reconstruction technique that incorporates additional DL super-resolution processing was recently introduced as a new type of DL-based reconstruction methodology; more specifically, several groups have described the use of model-based DL processing in the image reconstruction cycle, followed by the post-processing DL technique with a super-resolution function as an effective combination for DL-based image reconstruction [11–13]. This dual-type DL reconstruction including a super-resolution function has the potential to provide well-balanced images with a high SNR, high spatial resolution, and reasonable scan times, thus contributing to the acquisition of diagnostic images even in challenging body regions such as the head and neck.

We conducted the present study to assess the utility of dual-type DL-based image reconstruction with DL-based image denoising followed by super-resolution in a comparison with images that were reconstructed with the conventional parallel imaging-based method in head and neck Fs-T2WI.

## Materials and methods

### Patients

**Fig. 1:**
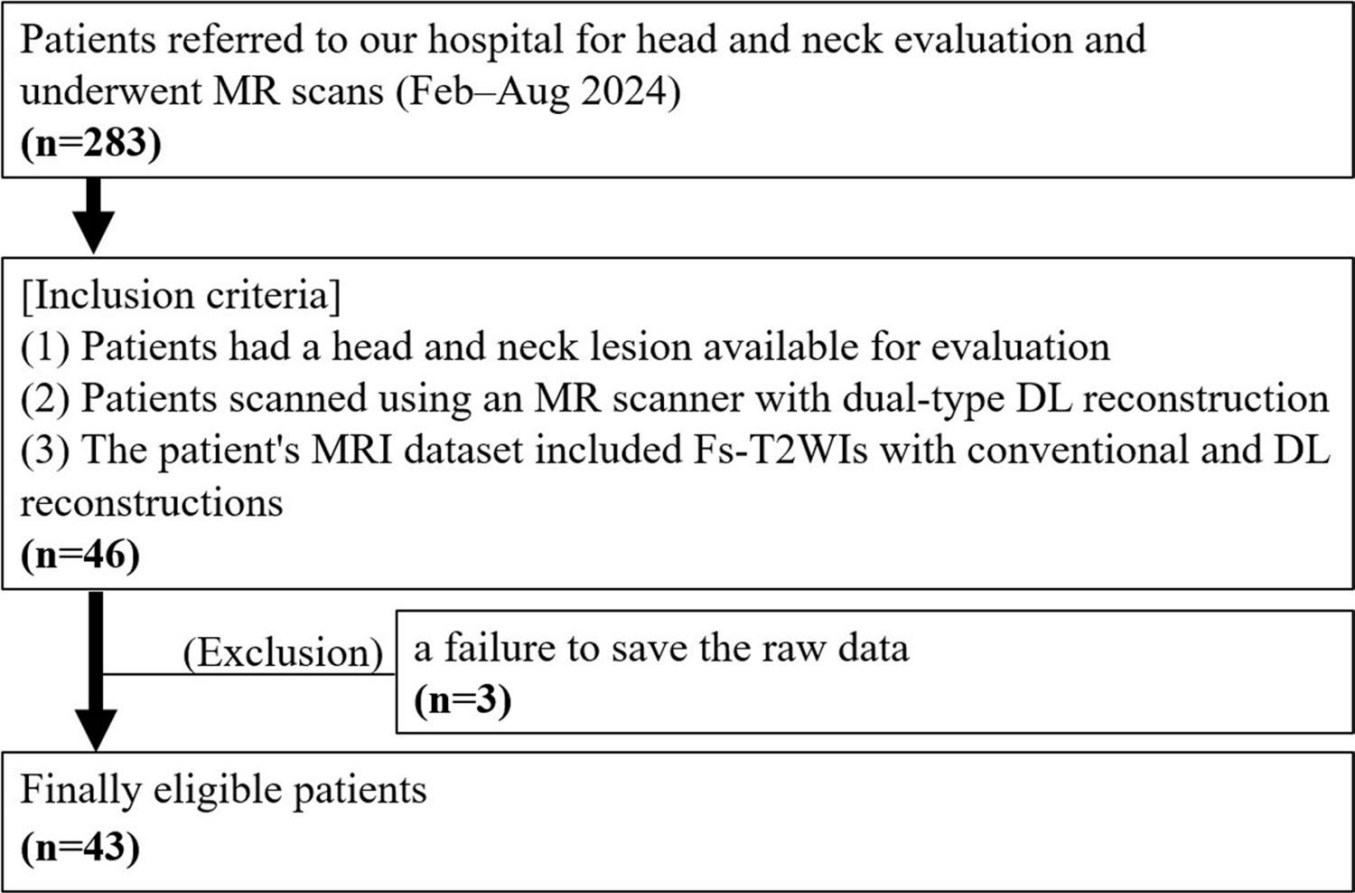
Flow diagram of the study population. *DL* deep learning; *Fs-T2WI* fat-suppressed T2-weighted imaging

The protocol of this retrospective study was approved by our institutional review board, and the requirement for patients’ written informed consent was waived. From February 2024 to August 2024, a total of 283 patients were referred to our hospital for a head and neck evaluation and underwent MR scanning. We selected the cases of 46 of these patients based on the following inclusion criteria: (1) the patient had a head and neck lesion available for evaluation and (2) had undergone the scanning by a specific MR scanner equipped with the dual-type DL-based image reconstruction function, and (3) the patient’s MRI dataset including Fs-T2WIs with both the conventional and DL-based image reconstructions was available. Three of these 46 patients were excluded based on a failure to save the raw data of Fs-T2WI for the image reconstruction. We analyzed the cases of the final total of 43 patients. Figure 1 depicts process of patient selection.

### Imaging parameters

All scanning was performed using a 3.0-Tesla MR unit (Ingenia Elition; Philips Healthcare, Best, Netherlands) with a 16-channel neurovascular coil. Two datasets of Fs-T2WI were respectively acquired: (1) 2D fast spin echo (FSE)-based FsT2WI with conventional image reconstruction, and (2) 2D FSE-based FsT2WI with dual-type DL-based image reconstruction. In both acquisitions, spectral adiabatic inversion recovery (SPAIR) was used for fat suppression. The craniocaudal scanning range was determined to place the target lesion at the center of the range. The details of the imaging parameters used for the two acquisitions are presented in Table 1. Note that the acquisition matrix for the DL-based T2WI was set to a value reduced to around 70% of that of the conventional T2WI; this setting of spatial resolution was determined based on previous studies, which investigated the utility of the super-resolution reconstruction technique in MRI, using a lower spatial resolution of approximately 65–80% compared to the conventional imaging protocol [11–13]. The acquisition time was, thus, shortened accordingly (scan time: 2 min 23 s in the conventional image reconstruction, and 1 min 36 s in the DL-based image reconstruction).

**Table 1:** The imaging parameters used in the acquisition by the two methods

	Conventional	DL
Repetition time: TR (ms)	4000	4000
Echo time: TE (ms)	97	97
Field of view (mm)	220 × 220	220 × 220
Acquisition matrix	416 × 287	292 × 188
Reconstruction matrix	512 × 512	640 × 640
Slice thickness (mm)	3.0	3.0
Inter-slice gap (mm)	0.9	0.9
Flip angle (degrees)	90	90
Reduction factor	1.6	1.6
Number of slice	27	27
Scan time	2 min 23 s	1 min 36 s

DL: deep learning

### Data processing

We used the dual-type DL framework in which two convolutional neural networks (CNNs) were working independently in the image reconstruction process. We first used the reconstruction model incorporating the DL architecture of Adaptive-CS-Net into the compressed-sensing sensitivity-encoding (Compressed SENSE)-based iterative denoising cycle, referred to hereafter as ‘model-based type DL reconstruction.’ Adaptive-CS-Net replaces the wavelet transform with the sparsifying transform in the CS denoising cycle. Effective image denoising was expected from this CNN-based method with the Adaptive-CS-Net-based sparsifying approach with the image reconstruction of a CS denoising cycle. More details of Adaptive-CS-Net and its denoising process have been described [14]. The image data were then transferred to SuperRes-Net, which was used as the second network in this model; this model was trained with multiple pairs of originally high-resolution and secondary downscaled images with Gibbs ringing artifacts for the upscaling of image resolution and the removal of Gibbs ringing artifacts. This neural network-based process was performed in the residual mode, which is commonly used for super-resolution solutions; it repeatedly applies a sequence that includes a 2D convolutional layer followed by a rectifier layer [15]. The dual-type DL-based reconstruction used in the present study incorporates technology from vendor prototypes, and all image processing was conducted within the MR console.

In contrast, the conventional image reconstruction consisted of sensitivity encoding (SENSE), which is one of the traditionally used methods for parallel imaging, and the zero-filling interpolation (ZIP) for the post-processing. The same reduction factor was used in the conventional and DL-based image reconstructions.

### Image analysis: qualitative assessment

As a qualitative assessment, two board-certified radiologists with 13 and 18 years of experience in head and neck imaging, respectively, visually evaluated the axial Fs-T2WIs with conventional- and DL-based reconstruction in a blinded fashion, focusing on: (*i*) the overall image quality, (*ii*) the visibility of anatomical structures, (*iii*) the degree of artifacts, (*iv*) lesion conspicuity, and (*v*) visibility of lesion edge. In the evaluation of ‘the visibility of anatomical structures,’ major anatomical structures in the neck, including visible muscles, the pharyngeal wall (pharyngeal mucosa and palatine tonsil), tongue, major salivary glands, lymph nodes, and bone, were each evaluated within the scan range. Each evaluation was conducted based on a five-point Likert scale as follows: 1 point, very poor; unavailable for diagnostic use; 2 points, poor, but possible for diagnostic use; 3 points, moderate, acceptable for diagnostic use; 4 points, good, minimal limitations for diagnostic use; and 5 points, excellent, almost no limitations for diagnostic use.

### Image analysis: quantitative assessment

In the quantitative assessment, the signal intensity of the lesion in each patient was measured by placing a polygonal region of interest (ROI) on an axial slice. If the lesion extended into two or more slices, the slice in which the largest area of lesion was depicted was selected. Any necrotic, cystic, or vessel component in the lesion was carefully excluded from the ROI. Another 1.0-cm-dia. ROI was placed on the muscle adjacent or nearest to the lesion while avoiding the signal intensity of blood vessels, noise, and artifact(s).

Both the polygonal ROI delineation of the lesion and the round ROI placement on the muscle were first performed on Fs-T2WIs with conventional image reconstruction, and then the ROI was copied onto T2WIs with DL-based image reconstruction, with the same ROIs placed at the same locations. If bulk noise (which can cause measurement bias) was present in any of the ROIs, the ROI’s shape and location were modified by referring to both the conventional and DL-based T2WIs to exclude the bulk noise.

The SNRs of the lesion were calculated as the mean signal in the lesion ROI divided by the standard deviation (SD) of the muscle ROI. For the contrast-to-noise ratio (CNR) measurement, we calculated the value of the difference between the mean signal of the lesion and the muscle ROI divided by the SD of the adjacent muscle’s ROI. The quantitative procedures were performed by a radiologist with 18 years of experience.

### Statistical analyses

Weighted Kappa statistics were used to determine the interobserver agreement for the qualitative analyses (0.00–0.20: poor, 0.21–0.40: fair, 0.41–0.60: moderate, 0.61–0.80: good, 0.81–1.00: excellent). Qualitative image scores were compared between the Fs-T2WI results with conventional and DL-based image reconstructions by the Wilcoxon signed-rank test. The SNRs of the lesions and the CNRs of the lesion to a muscle were, respectively, compared between the Fs-T2WI with conventional and DL-based image reconstruction using the paired *t* test, after the confirmation of the data’s normal distribution by the Shapiro–Wilk test. A *p* value < 0.05 was considered significant. SPSS software (IBM, Armonk, NY) was used for all statistical analyses.

## Results

The characteristics of the 43 patients were as follows: 26 males and 17 females, median age 58 yrs (range 32–76 yrs). The primary lesions revealed by their MRI examinations were as follows: orbital tumor (*n* = 4 patients: malignant lymphoma in three, and schwannoma in one), nasal or paranasal cavity tumor (*n* = 13: squamous cell carcinoma in seven, inverted papilloma in five, and glomangiopericytoma in one), facial or neck soft tissue lesion (*n* = 9: epidermoid in three, schwannoma in three, hemangioma in one, myofibroblastic tumor in one, and basal cell carcinoma in one), parotid gland tumor (*n* = 12: pleomorphic adenoma in eight, Warthin tumor in three, and salivary duct carcinoma in one), oral cavity tumor (*n* = 3: all had squamous cell carcinoma), and pharyngeal tumor (*n* = 2: all had squamous cell carcinoma). The patients’ characteristics are summarized in Table 2.

**Table 2:** The characteristics of the study cohort (43 patients who underwent MRI for head and neck lesions)

Total patients (*n* = 43)	
Age	
Range	32–76
Median	58
Gender	
Male	26
Female	17
Detail of primary lesion	
Orbital tumor	4
Lymphoma	3
Schwannoma	1
Nasal or sinonasal cavity tumor	13
Squamous cell carcinoma	7
Inverted papilloma	5
Glomangiopericytoma	1
Facial or neck soft tissue lesion	9
Schwannoma	3
Epidermoid	3
Hemangioma	1
Myofibroblastic tumor	1
Basal cell carcinoma	1
Parotid gland tumor	12
Pleomorphic adenoma	8
Warthin tumor	3
Salivary duct carcinoma	1
Oral cavity tumor	3
Squamous cell carcinoma	3
Pharyngeal tumor	2
Squamous cell carcinoma	2

In the qualitative analysis, significant differences were observed between the Fs-T2WI with the conventional and DL-based image reconstructions in all of the evaluation items except the degree of artifacts. The results of the qualitative analysis are summarized in Table 3. The interobserver agreement in all of the qualitative analyses between the two board-certified radiologists was mostly good (weighted Kappa scores 0.57–0.71).

**Table 3:** Results of the qualitative assessment

	Reader 1			Reader 2			Kappa score
	Conventional	DL	p value	Conventional	DL	*p* value	
Overall image quality	3.15 ± 0.46	3.86 ± 0.34	< 0.001	3.15 ± 0.47	3.82 ± 0.38	< 0.001	0.71
Visibility of anatomical structures	3.17 ± 0.43	3.93 ± 0.24	< 0.001	3.08 ± 0.55	3.97 ± 0.39	< 0.001	0.61
Degree of the artifact	3.17 ± 0.61	3.32 ± 0.63	0.09	3.35 ± 0.60	3.46 ± 0.66	0.16	0.57
Lesion conspicuity	3.45 ± 0.62	3.82 ± 0.43	< 0.001	3.44 ± 0.62	3.88 ± 0.53	< 0.001	0.62
Visibility of lesion edge	2.97 ± 0.33	3.90 ± 0.31	< 0.001	3.13 ± 0.40	4.04 ± 0.47	< 0.001	0.64

Data are mean ± standard deviation

**Fig. 2:**
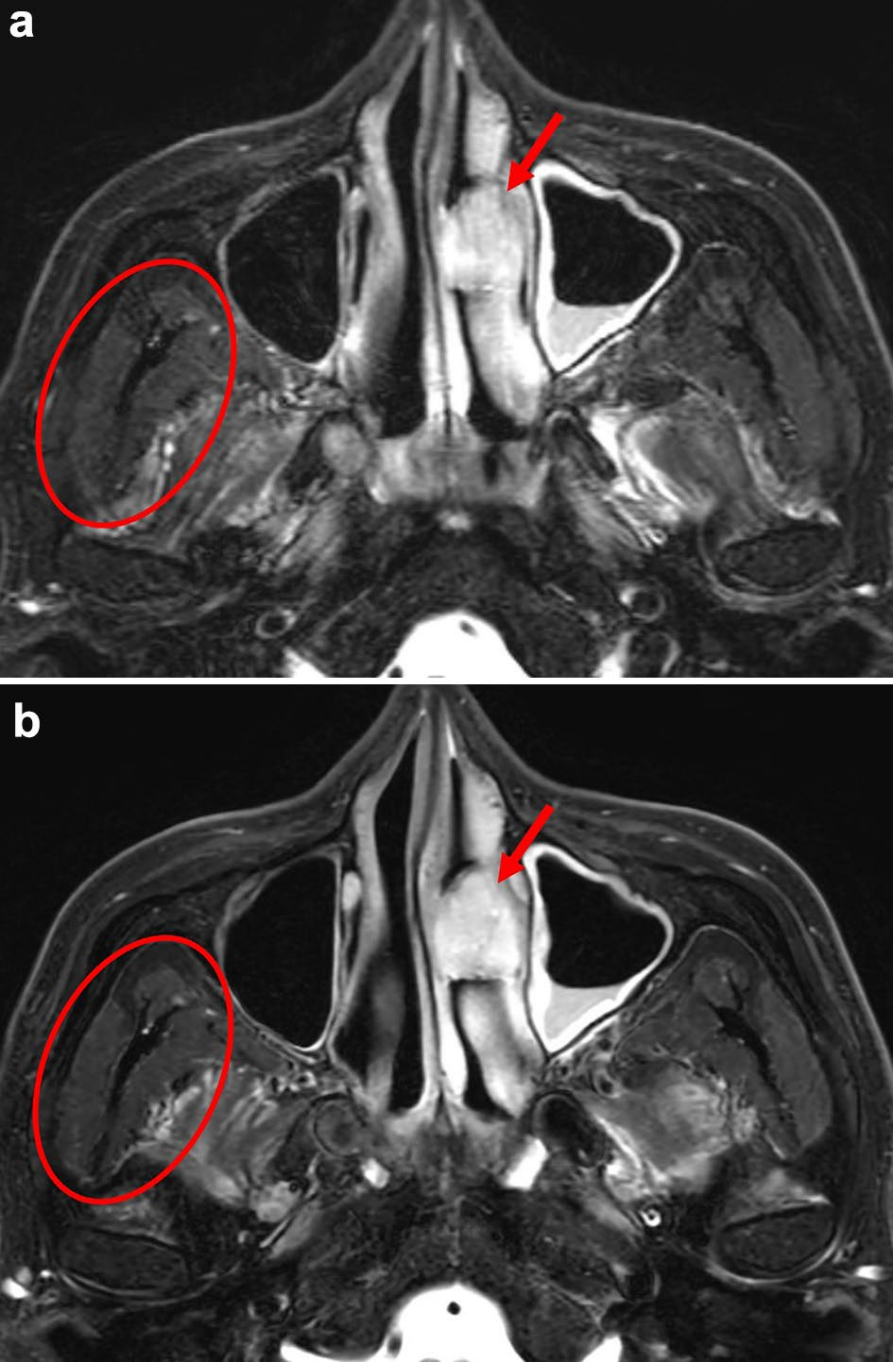
Representative patient with a nasal cavity tumor. Fs-T2WIs with conventional image reconstruction (**a**) and DL-based image reconstruction (**b**) in the patient. Overall, the visibility of normal structures was considered more prominent in the DL-based Fs-T2WI compared to the conventional method, e.g., in muscles with improved edge sharpness (a,b: *circles*). The tumor was observed more conspicuously in DL-based Fs-T2WI with reduced artifacts compared to the conventional method-based Fs-T2WI (a,b: *arrows*)

**Fig. 3:**
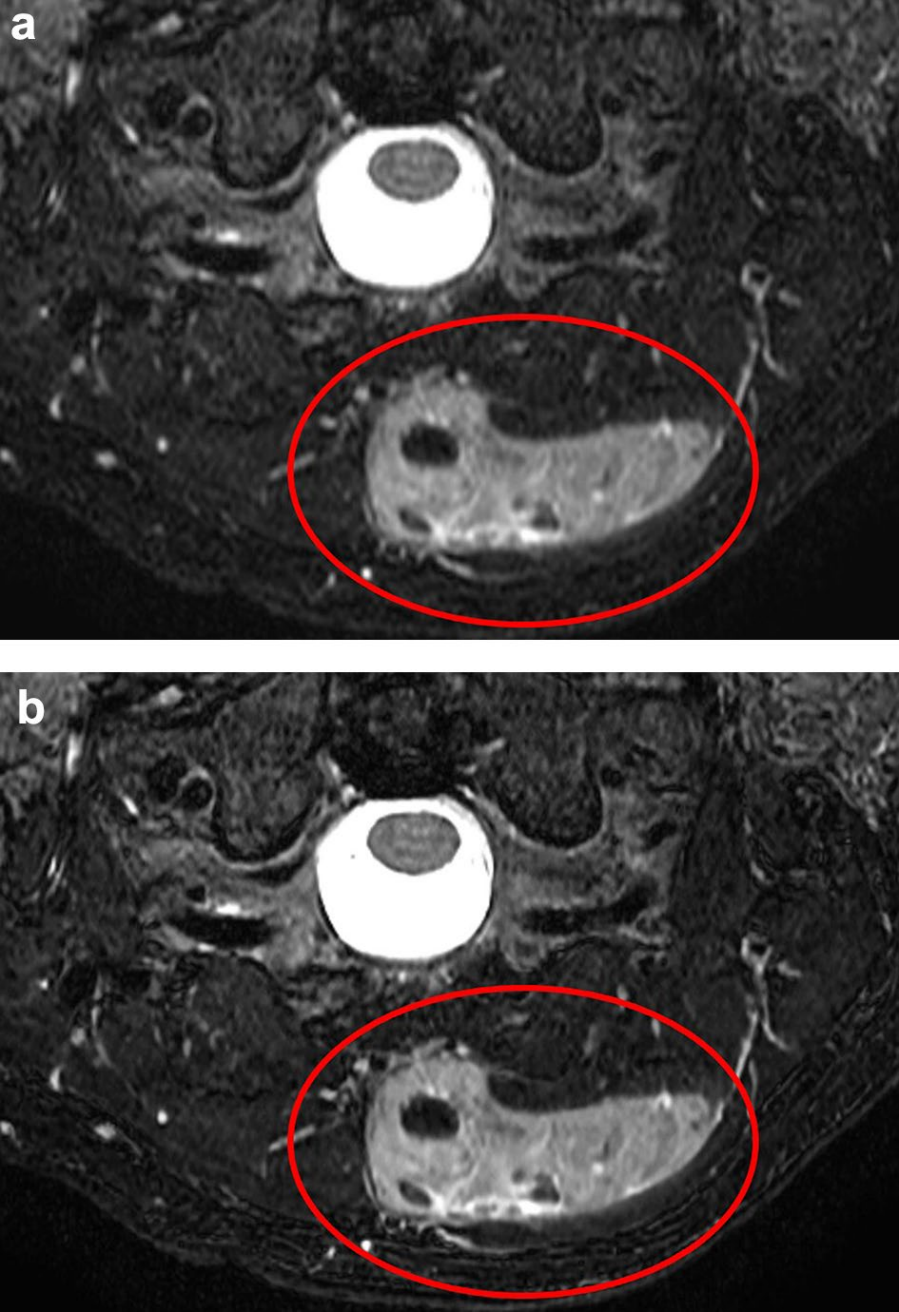
Representative patient with a neck soft tissue tumor. Fs-T2WIs obtained with conventional image reconstruction (**a**) and DL-based image reconstruction (**b**) in the patient. The visibility of the lesion edges was markedly improved in the DL-based Fs-T2WI compared to the conventional approach (a,b: *circles*)

**Fig. 4:**
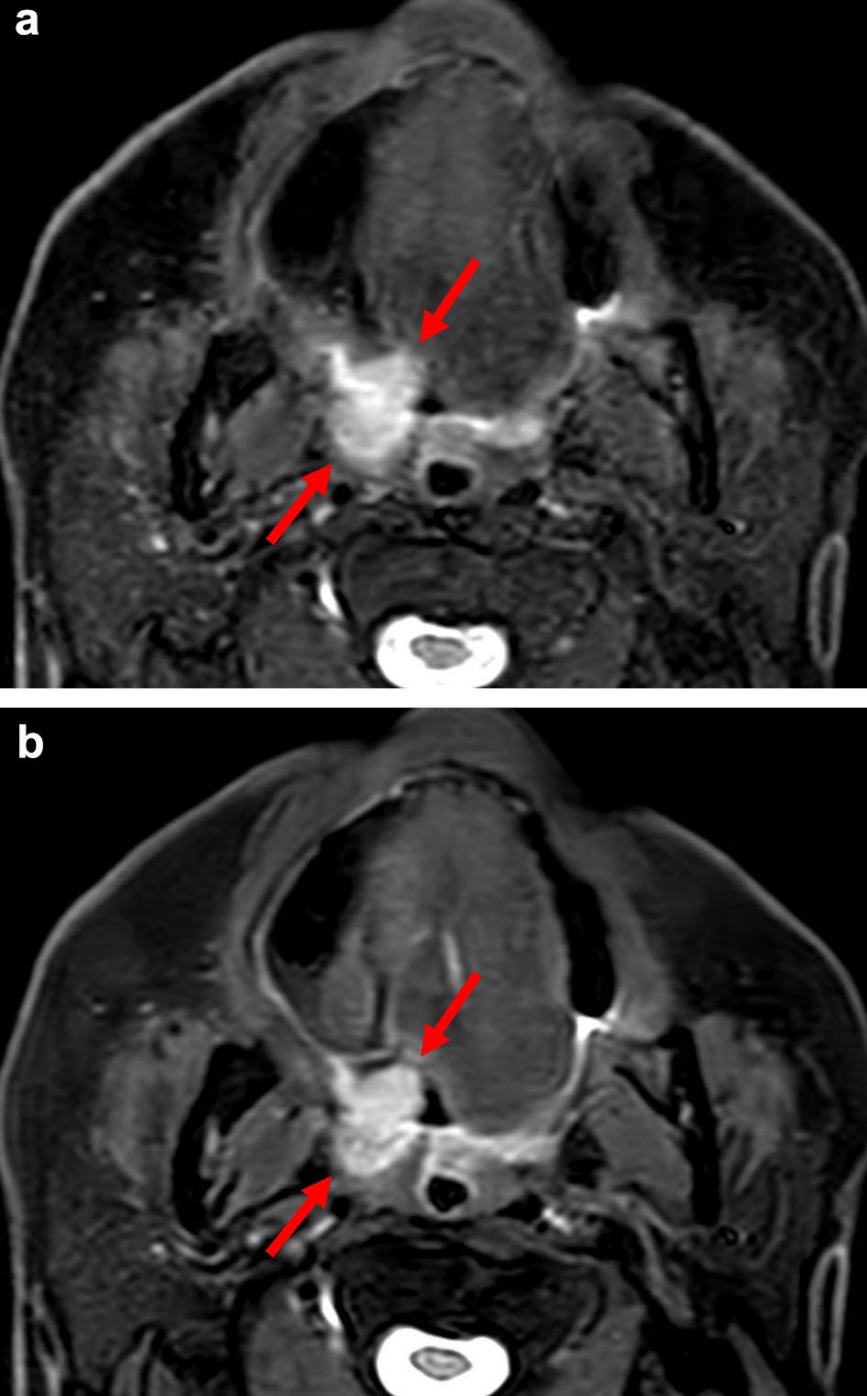
Representative patient with an oropharyngeal squamous cell carcinoma. Fs-T2WIs obtained with conventional image reconstruction (**a**) and DL-based image reconstruction (**b**) in the patient. In the conventional image, lesion edge visibility was somewhat unclear, likely due to image noise or blurring (a: *arrows*). The DL-based image offered clear lesion conspicuity and edge visibility (b: *arrows*). Furthermore, it provided high overall quality, ensuring clear visualization of normal anatomical structures as well as the lesion

**Fig. 5:**
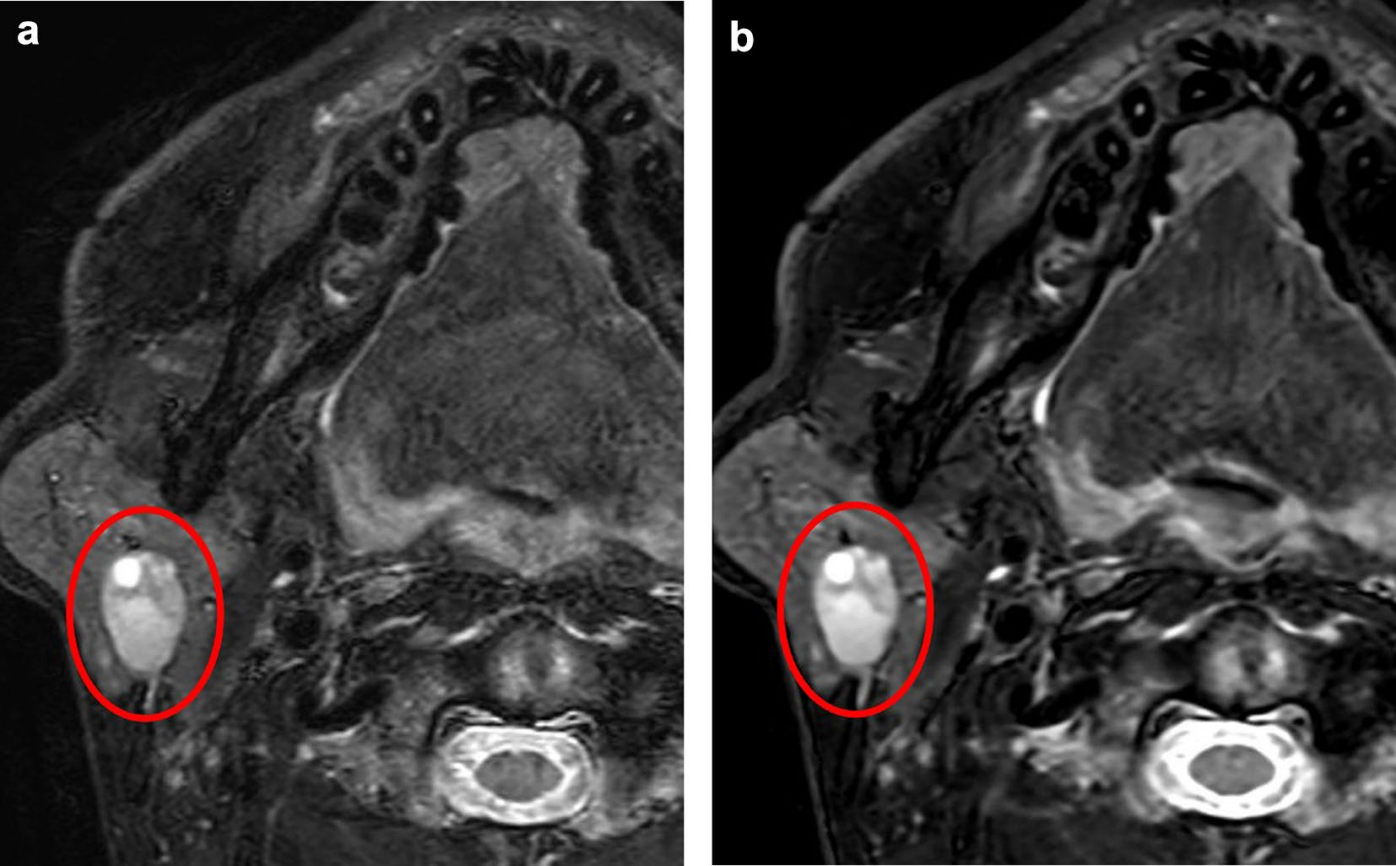
Representative patient with a parotid gland tumor. Fs-T2WIs acquired with conventional image reconstruction (**a**) and DL-based image reconstruction (**b**) in the patient. In the DL-based Fs-T2WI, nearly all normal anatomical structures (e.g., the base of the tongue, palatine tonsil, muscles, and parotid gland) were relatively clearly visible with less noise compared to the conventional method. Moreover, the parotid gland tumor exhibited slightly improved lesion conspicuity and edge visibility in the DL-based Fs-T2WI (a,b: *circles*)

In the quantitative analysis, the mean ± SD size of the ROIs for the delineation of the patients; lesions was 312 ± 173 mm^2^. All of the quantitative values were observed to follow a normal distribution based on the Shapiro–Wilk test. Significant differences were observed in the SNR between the Fs-T2WI with conventional reconstruction (21.4 ± 14.7) and the DL-based image reconstruction (26.2 ± 13.5) (*p* < 0.001). In the CNR assessment, the CNR between the lesion and adjacent or nearest muscle in the DL-based Fs-T2WI (16.8 ± 11.6) was significantly higher than that in the conventional Fs-T2WI (14.2 ± 12.9) (*p* < 0.001). Table 4 provides the results of all the quantitative analyses, and Figs. 2, 3, 4 and 5 present the Fs-T2WI results obtained for representative patients by conventional and DL-based image reconstruction.

**Table 4:** Results of the quantitative assessment

	Conventional	DL	*p* value	
SNR	21.4 ± 14.7	26.2 ± 13.5	< 0.001	
CNR	14.2 ± 12.9	16.8 ± 11.6	< 0.001	

Data are mean ± standard deviation. *DL* deep learning, *SNR* signal to noise ratio, *CNR* contrast to noise ratio

## Discussion

These results demonstrate that for evaluations of the head and neck, Fs-T2WI with the dual-type DL-based image reconstruction technique with Adaptive-CS-Net and Super-ResNet successfully improved the image quality in both quantitative and qualitative assessments within shortened acquisition times compared to the use of conventional image reconstruction. Our search of the relevant literature indicates that the present study provides the first result regarding the effectiveness of dual-type DL-based image reconstruction with image denoising and super-resolution processing in head and neck MRI. This image reconstruction technique has the potential to contribute to daily clinical radiology practice for the assessment of the head and neck by providing high SNRs, CNRs, and spatial resolution. We evaluated various types of head and neck lesions at varying anatomical sites for the quantitative and qualitative assessments, and improved image quality was obtained, suggesting that the new method described herein can provide excellent image quality with high spatial resolution even within reduced acquisition times for overall evaluations of the head and neck in routine clinical practice.

T2WI with conventional parallel imaging-based FSE sequence has been a commonly used method in recent decades and has frequently been applied for evaluations of the head and neck. However, with such a conventional method, the acquisition time is often long when the spatial resolution is set sufficiently high, and the SNR is sometimes insufficient. Although compressed sensing has been described as an effective method to reduce the scan time without decreasing the image quality and has been applied in the head and neck T2WI sequence [7], the random sampling pattern in the CS technique is somewhat difficult to effectively apply in a 2D sequence, whereas the 3D-T2WI sequence is somewhat challenging for routine clinical scans due to its long scan time.

A DL reconstruction technique was recently introduced as an effective method for denoising and an additional MR reconstruction tools [16, 17], and several investigations have indicated the utility of DL techniques for head and neck MRI [18–20]. However, although image quality improvement was achieved in the high-spatial-resolution images that were required for the head and neck region, the scan time was still relatively long, making it challenging to achieve a reduction in the scan time and an improvement in image quality simultaneously. In contrast, dual-type DL-based image reconstruction with the combination of denoising and super-resolution successfully achieved both high image quality and high spatial resolution within a shortened image acquisition time.

Several studies have investigated DL-based image quality improvement in the acquisition of the head and neck MRI [18–20]. The image denoising in those studies was performed mainly using a single CNN-based model. Our investigation fully utilized the dual-type DL model with the effective combination of the Adaptive-CS-Net and Super-Res Net. Adaptive-CS-Net is based on the model-based-type of DL image reconstruction in which the CNN is embedded into the iterative denoising cycle of the CS reconstruction process. In this model, a large amount of signal data during the image acquisition and processing can be well utilized and handled for effective denoising [14, 21]. In addition, the second process of the dual-type DL model used herein adopted Super-Res Net reconstruction to obtain images with higher spatial resolution. Notably, we applied a 30% reduction of the acquired spatial resolution, and thereafter the matrix was sufficiently elevated with the super-resolution reconstruction technique, resulting in improved image quality with high spatial resolution even within the reduced scan time afforded by the lower acquired matrix. This well-balanced acquisition setting resulted in images excelling in all aspects: a high SNR and a high CNR with effective denoising, high spatial resolution with edges sharpness, and reduced scan times.

Our study has several limitations. The number of patients was small (*n* = 43) and drawn from a single institution. Our investigation should, thus, be treated as a preliminary result. Particularly, in the current study, a significant difference was not observed only in the qualitative evaluation of the ‘degree of artifact.’ Because the shortened scan time is expected to reduce motion artifacts, further investigation with an increased number of patients is likely to provide statistically significant findings related to the degree of artifact. Next, we did not use the ground truth data for the image evaluation. High-SNR and high-spatial-resolution images acquired with very long scan times are generally used for the ground truth, but head and neck MRI examinations often suffer from patient-motion artifacts, and thus images derived from long scan times are not necessarily high-quality and may be inappropriate for the ground truth. Furthermore, we did not conduct an investigation to determine which spatial resolution (e.g., 65%, 70%, 75%, or 80% compared to the standard spatial resolution in conventional images) would be optimal for super-resolution reconstruction, as this remains an issue for the additional investigation. In addition, the locations of the patients’ lesions were somewhat biased toward maxillofacial and orbital lesions. An additional number of patients with lesions at other head and neck sites such as the pharynx and larynx should be assessed in the future analysis.

In conclusion, dual-type DL-based image reconstruction by effective denoising and a super-resolution process successfully provided high image quality in head and neck Fs-T2WI with shortened scan times compared to conventional image reconstruction. This method can be a useful tool for the assessment of patients with head and neck lesions.

## References

[1] Christe A, Waldherr C, Hallett R, Zbaeren P, Thoeny H. MR imaging of parotid tumors: typical lesion characteristics in MR imaging improve discrimination between benign and malignant disease. AJNR Am J Neuroradiol. 2011;32:1202–7. 21724574 10.3174/ajnr.A2520 PMC7966029

[2] Saat R, Mahmood G, Laulajainen-Hongisto A, Lempinen L, Aarnisalo AA, Jero J, et al. Comparison of MR imaging findings in paediatric and adult patients with acute mastoiditis and incidental intramastoid bright signal on T2-weighted images. Eur Radiol. 2016;26:2632–9. 26607577 10.1007/s00330-015-4113-5

[3] Li Z, Wang X, Jiang H, Qu X, Wang C, Chen X, et al. Chronic invasive fungal rhinosinusitis vs sinonasal squamous cell carcinoma: the differentiating value of MRI. Eur Radiol. 2020;30:4466–74. 32279114 10.1007/s00330-020-06838-1

[4] Akutsu A, Horikoshi T, Yokota H, Wada T, Motoori K, Nasu K, et al. MR imaging findings of carcinoma ex pleomorphic adenoma related to extracapsular invasion and prognosis. AJNR Am J Neuroradiol. 2022;43:1639–45. 36202554 10.3174/ajnr.A7656 PMC9731259

[5] Touska P, Connor SEJ. Recent advances in MRI of the head and neck, skull base and cranial nerves: new and evolving sequences, analyses and clinical applications. Br J Radiol. 2019;92:20190513. 31529977 10.1259/bjr.20190513 PMC6913354

[6] Takumi K, Nagano H, Nakanosono R, Kumagae Y, Fukukura Y, Yoshiura T. Combined signal averaging and compressed sensing: impact on quality of contrast-enhanced fat-suppressed 3D turbo field-echo imaging for pharyngolaryngeal squamous cell carcinoma. Neuroradiology. 2020;62:1293–9. 32577772 10.1007/s00234-020-02480-2

[7] Tomita H, Deguchi Y, Fukuchi H, Fujikawa A, Kurihara Y, Kitsukawa K, et al. Combination of compressed sensing and parallel imaging for T2-weighted imaging of the oral cavity in healthy volunteers: comparison with parallel imaging. Eur Radiol. 2021;31:6305–11. 33517492 10.1007/s00330-021-07699-y

[8] Kami Y, Chikui T, Togao O, Kawano S, Fujii S, Ooga M, et al. Usefulness of reconstructed images of Gd-enhanced 3D gradient echo sequences with compressed sensing for mandibular cancer diagnosis: comparison with CT images and histopathological findings. Eur Radiol. 2023;33:845–53. 35986770 10.1007/s00330-022-09075-w

[9] Kami Y, Chikui T, Togao O, Ooga M, Yoshiura K. Comparison of image quality of head and neck lesions between 3D gradient echo sequences with compressed sensing and the multi-slice spin echo sequence. Acta Radiol Open. 2020;9:2058460120956644. 35140985 10.1177/2058460120956644 PMC8819772

[10] Fujima N, Kamagata K, Ueda D, Fujita S, Fushimi Y, Yanagawa M, et al. Current state of artificial intelligence in clinical applications for head and neck MR imaging. Magn Reson Med Sci. 2023;22:401–14. 37532584 10.2463/mrms.rev.2023-0047 PMC10552661

[11] Bischoff LM, Peeters JM, Weinhold L, Krausewitz P, Ellinger J, Katemann C, et al. Deep learning super-resolution reconstruction for fast and motion-robust T2-weighted prostate MRI. Radiology. 2023;308: e230427. 37750774 10.1148/radiol.230427

[12] Terzis R, Dratsch T, Hahnfeldt R, Basten L, Rauen P, Sonnabend K, et al. Five-minute knee MRI: An AI-based super resolution reconstruction approach for compressed sensing A validation study on healthy volunteers. Eur J Radiol. 2024;175: 111418. 10.1016/j.ejrad.2024.111418 38490130

[13] Kravchenko D, Isaak A, Mesropyan N, Peeters JM, Kuetting D, Pieper CC, et al. Deep learning super-resolution reconstruction for fast and high-quality cine cardiovascular magnetic resonance. Eur Radiol. 2024. 10.1007/s00330-024-11145-0 10.1007/s00330-024-11145-0 PMC12021735 39441391

[14] Pezzotti N, Yousefi S, Elmahdy MS, Van Gemert JHF, Schuelke C, Doneva M, et al. An Adaptive Intelligence Algorithm for Undersampled Knee MRI Reconstruction. IEEE Access. 2020;8:204825–38.

[15] Chaudhari AS, Fang Z, Kogan F, Wood J, Stevens KJ, Gibbons EK, et al. Super-resolution musculoskeletal MRI using deep learning. Magn Reson Med. 2018;80:2139–54. 29582464 10.1002/mrm.27178 PMC6107420

[16] Lin DJ, Johnson PM, Knoll F, Lui YW. Artificial intelligence for MR image reconstruction: an overview for clinicians. J Magn Reson Imaging. 2021;53:1015–28. 32048372 10.1002/jmri.27078 PMC7423636

[17] Heckel R, Jacob M, Chaudhari A, Perlman O, Shimron E. Deep learning for accelerated and robust MRI reconstruction. MAGMA. 2024;37:335–68. 39042206 10.1007/s10334-024-01173-8 PMC11316714

[18] Liu H, Deng D, Zeng W, Huang Y, Zheng C, Li X, et al. AI-assisted compressed sensing and parallel imaging sequences for MRI of patients with nasopharyngeal carcinoma: comparison of their capabilities in terms of examination time and image quality. Eur Radiol. 2023;33:7686–96. 37219618 10.1007/s00330-023-09742-6 PMC10598173

[19] Fujima N, Nakagawa J, Kameda H, Ikebe Y, Harada T, Shimizu Y, et al. Improvement of image quality in diffusion-weighted imaging with model-based deep learning reconstruction for evaluations of the head and neck. MAGMA. 2023; 10.1007/s10334-023-01129-4 10.1007/s10334-023-01129-4 37989922

[20] Fujima N, Nakagawa J, Ikebe Y, Kameda H, Harada T, Shimizu Y, et al. Improved image quality in contrast-enhanced 3D–T1 weighted sequence by compressed sensing-based deep-learning reconstruction for the evaluation of head and neck. Magn Reson Imaging. 2024;108:111–5. 38340971 10.1016/j.mri.2024.02.006

[21] Foreman SC, Neumann J, Han J, Harrasser N, Weiss K, Peeters JM, et al. Deep learning-based acceleration of Compressed Sense MR imaging of the ankle. Eur Radiol. 2022;32:8376–85. 35751695 10.1007/s00330-022-08919-9 PMC9705492

